# Qualitative Evaluation of an Online Technology to Support Rural Caregivers of People with Dementia

**DOI:** 10.3390/geriatrics10060161

**Published:** 2025-12-05

**Authors:** Carmela Leone, Clare Wilding, Tshepo Rasekaba, Megan E. O’Connell, Debra Morgan, Irene Blackberry

**Affiliations:** 1 John Richards Centre for Rural Ageing Research, La Trobe Rural Health School, La Trobe University, Wodonga, VIC 3689, Australia; c.leone@latrobe.edu.au (C.L.); clare.wilding1@outlook.com (C.W.); t.rasekaba@latrobe.edu.au (T.R.); 2 Department of Psychology & Health Studies, University of Saskatchewan, Saskatoon, SK S7N 5A5, Canada; megan.oconnell@usask.ca; 3 Canadian Centre for Rural and Agricultural Health, University of Saskatchewan, Saskatoon, SK S7N 2Z4, Canada; debra.morgan@usask.ca; 4 Care Economy Research Institute, La Trobe University, Wodonga, VIC 3689, Australia

**Keywords:** dementia, caregiver, community, mobile application, rural, virtual, dementia-friendly

## Abstract

**Background/Objectives**: In rural communities, caregivers of people living with dementia face limited access to support services. Digital interventions offer potential solutions for support. This paper reports on the evaluation of Verily Connect, a web-based multicomponent intervention developed to support caregivers. The aim of this qualitative study was to critically evaluate the implementation of Verily Connect to better understand its barriers and enablers. **Methods**: Using the Consolidated Framework for Implementation Research (CFIR), qualitative data were collected through semi-structured interviews with 24 health service professionals across 12 rural Australian communities. Thematic analysis was conducted to identify barriers and facilitators to implementation. **Results**: Key barriers included limited digital literacy, resistance to technology and privacy concerns, as well as competing organisational priorities, and inadequate technological infrastructure. Facilitators included organisational alignment and supportive management. **Conclusions**: The perceived relevance and usability of Verily Connect were enhanced by its co-design with caregivers and integration into health service models. Addressing digital literacy for caregivers, infrastructure limitations, and organisational readiness is essential for future technology-based health interventions in rural dementia care.

## 1. Introduction

Most people living with dementia live in their communities, where they are cared for by a family member [1,2]. Informal caregivers/carers provide a substantial amount of time caring [1,3], performing a variety of duties [4,5]. Role demands increase as dementia symptoms progress [6], and caregivers often experience psychological distress [7,8] as well as depression, anxiety, and burden [9,10,11,12].

Caregiver interventions can mitigate the psychological consequences of caregiving [13]. Support group interventions increase caregiver social support and decrease depression and anxiety [14]. Multicomponent interventions are considered most effective because they impact multiple possible outcomes [15]. The location of intervention matters: healthcare interventions developed for urban residents but delivered to rural residents are often ineffective [16,17,18]. Customisation of interventions for the rural context is a key facilitator to reducing disparities in rural dementia care [19].

In comparison to their urban counterparts, rural-dwelling caregivers provide a higher degree of caregiving, are less likely to have their own healthcare needs met [20] and have less access to formal supports [21]. Limited access to support, coupled with the geographical isolation of rural communities, can exacerbate the impact of supporting a person living with dementia, and lead to a reliance on informal networks [22]. However, online technologies can enhance social support for rural caregivers of people living with dementia [23]. Rural residents also have reduced access to healthcare services and consequently experience relatively poorer health [24]. Inequities in healthcare access for rural residents can be overcome using technology [23].

Rural families, however, have less access to technology, which is the digital divide [25]. Physical access barriers are accompanied by psychological barriers, such as lack of confidence, which persist even if the physical access barriers can be removed [26]. Implementation of an intervention in the rural context must consider the barriers to use of technology experienced by rural residents [27], including limited digital literacy and a lack of trust which can significantly affect its uptake and effectiveness [26]. Interventions designed for rural caregivers must therefore not only provide support but also incorporate strategies to overcome these barriers.

Technology-based interventions for dementia caregivers have demonstrated benefits in improving well-being, with evidence from online programs, videophone support, and web-based social platforms [28,29,30,31,32]. However, there is limited understanding of the contextual factors that influence adoption, particularly in rural settings where caregivers face unique challenges. In rural Australia, where communities experience significant digital inequities, addressing these challenges is critical.

Virtual multicomponent interventions that are co-designed with rural communities, such as Virtual Dementia Friendly Rural Community—Verily Connect [33], address these challenges by combining education, resource navigation, and social support in a format tailored for rural contexts. Verily Connect has the following multiple features: (1) educational Guides that were co-created by the research team and caregivers; (2) local in-person Resources accessible from each rural community, resources that require some travel to access, and online resources curated by the research team; (3) private and secure group Chat forums; and (4) videoconference support groups [33]. The aim of this qualitative study was to critically evaluate the implementation of Verily Connect to better understand its barriers and enablers.

## 2. Materials and Methods

### 2.1. Study Design

The implementation of Verily Connect was evaluated using the Consolidated Framework of Implementation Research (CFIR) [34] as a theoretical framework and a thematic analysis method [35]. Qualitative data were collected using a semi-structured interview method.

The current study is part of, and complements, a larger study which used mixed-methods and a stepped-wedge, cluster-randomised controlled trial design to evaluate the effectiveness and implementation of Verily Connect [33]. The aims of the Verily Connect website and application include increasing support for the caregivers of people living with dementia in rural communities and strengthening communities to become dementia-friendly [33]. The full study protocol for the larger study is published elsewhere [36]. The study described in this paper used qualitative data collected from interviews with health service professionals who trialled Verily Connect between 2018 and 2019, collecting their feedback and perspectives about its implementation in the community and the organisations in which they worked. Because this study’s focus is on implementation rather than user perspectives, health service professionals were selected for their role in integrating Verily Connect into service workflows.

### 2.2. Ethics Approval

Ethical and legal requirements outlined in the National Statement on Ethical Conduct on Human Research [37] were adhered to, and the study followed the principles of the Helsinki Declaration. Approval for the research was granted by the Melbourne Health Human Research Ethics Committee (HREC/17/MH/404, reference 2017.376).

### 2.3. Study Setting

Participants were recruited from 12 rural communities in Australia: eight communities from the state of Victoria, two from New South Wales, and two from South Australia. These states share borders and are located in south-eastern Australia. For each of the participating communities, a local rural health service partner was identified, from which staff members were recruited.

### 2.4. Recruitment

Participants were purposively recruited via open community forums, and the full recruitment process is described in the study protocol [36]. A total of 37 health service staff volunteered to participate in the larger implementation of Verily Connect study, and 24 of those participants agreed to be and were interviewed for the study described in this paper. At the end of the implementation period, staff members were invited to participate in semi-structured interviews. All participants provided written consent. Inclusion criteria for service provider staff were

- The current provision of a dementia service or service for older adults in one of the catchment areas;
- Access to a smartphone or tablet, or computer with internet access.

### 2.5. Data Collection and Analysis

During the trial, staff members were asked to engage with the Verily Connect application for a minimum period of 10 min per engagement, using a computer or a mobile device, on at least 2 occasions. The duration of engagement was discussed between researchers, and 10 min was considered enough time for participants to fully engage with the application, without the task being too onerous. Both the computer web browser-accessible and mobile-accessible versions of Verily Connect provided the same content. After the end of the trial implementation period in November 2018, staff were invited to provide feedback via semi-structured interviews.

Interviews were conducted via phone or video conference in 2019, using an Interview Schedule (see Appendix A) which was developed by drawing from the question bank for the CFIR [38]. Participants each participated in one interview, the duration of which ranged from approximately 10 min to one hour. All participants who agreed to participate were interviewed.

The transcripts were imported into NVivo, and data were coded by the first and second authors using the online CFIR handbook [38]. In a second round of thematic analysis, data were further analysed to answer the research question: “What are the barriers to, and enablers of, the implementation of Verily Connect?” Thus, data were analysed deductively in the first instance, against the CFIR conceptual framework, and were analysed inductively to identify themes in a subsequent round of analysis. Two coders were used to strengthen rigour, and inter-coder reliability was maintained through a consensus process. Coding discrepancies were discussed in iterative meetings until agreement was reached, ensuring consistency in theme development, while facilitating reflexivity. Although these steps helped mitigate bias, the inherently subjective nature of thematic analysis means that findings should be considered within this limitation, as interpretations can be influenced by researchers’ perspectives and prior assumptions, even when guided by the CFIR framework.

## 3. Results

Several themes were identified, and an overview of the CFIR constructs and related themes is presented in Table 1. The themes are categorised as either a barrier to or facilitator of implementation.

**Figure 1:**
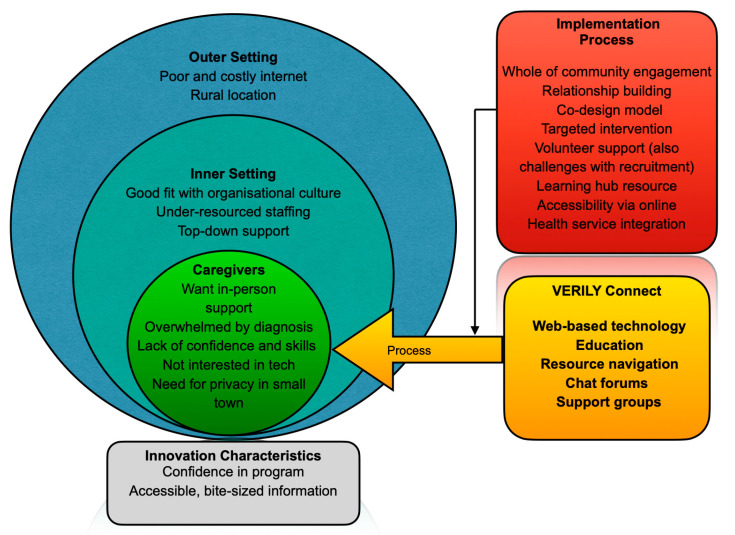
Lower-level themes.

Participants perceived that older people—as a group and as the anticipated users of the application—were indisposed to engaging with Verily Connect due to various Individual Characteristics: they lacked digital literacy and were disinterested in, or fearful of, using technology; they were concerned about privacy; they were feeling overwhelmed; and/or they preferred receiving face-to-face, one-on-one support rather than the online and group-based support provided by Verily Connect. The lack of sufficient dedicated staffing and competing priorities within the health services (Inner Setting), as well as challenges recruiting volunteers (Process), were perceived as organisational barriers to the implementation of Verily Connect, and the lack of infrastructure to support an online-based service (Outer Setting) was viewed as an environmental barrier. In contrast, there were four main factors that were seen to enhance the uptake of Verily Connect: the program’s alignment with health service goals (Inner Setting); the involvement of a university (Innovation Characteristics); good support of the program from management (Inner Setting); and that Verily Connect was easy to understand and had some effective marketing strategies (Innovation Characteristics). A visual representation of the lower-level themes is presented in Figure 1.

### 3.1. Innovation Characteristics

#### 3.1.1. Evidence Strength and Quality

##### Grounded in Research

Verily Connect was developed and implemented through a partnership of five universities, and this foundation in research contributed to trust and confidence in the program:

We had a strong level of trust in the information that was going to be provided, so that it was going to be accurate and reliable… I think that was something that we had a strong sense that this would be a good project to participate in. (HS7)

The evidence-based nature of the program was seen to provide assurance: *“I really love that it’s got the research behind it and that’s its great information”* (HS16). The fact that Verily Connect is grounded in research contributed to its perceived legitimacy and influenced its acceptance.

#### 3.1.2. Design Quality and Packaging

##### Supportive Information and Resources

The information provided by Verily Connect was perceived to be supportive and a positive aspect of the project, as was the way in which the information was provided: “*I think some of the resources, so like the postcards, were really good and easy to give to people*” (HS15). The positives included local services information, and accessibility for older people: “*The information when I’ve been on the app [application]… it’s really easy to read, well written*” (HS16). The information was also considered a good fit for a resource aimed at supporting caregivers:

We’re actually looking at carer engagement, carers’ handbooks and thinking about putting together a carer’s resource package… Verily would fit in nicely into that, where if there’s anyone with any sort of cognitive impairment, we’ll give them that information and it becomes part of the package. (HS15)

Clear, accessible and user-friendly information and resources that practically support the needs of caregivers were valued by the participants.

### 3.2. Outer Setting

#### 3.2.1. Needs and Resources

##### Resource and Infrastructure Challenges

Appropriate infrastructure is needed for online technology to be used effectively. However, in many of the rural communities, poor and costly access to the Internet limited the effectiveness of the Verily Connect implementation: “*The telecommunication coverage is patchy…. If you’ve got a poor community where you’ve got dodgy [internet provider] coverage, that’s probably not predisposed for people to take up a technology option*” (HS1). A lack of affordability in accessing computers and mobile equipment was also a barrier for some: “*Most people have smart phones and computers and iPads now, but for some community members who can’t afford it—because we do have a section who are from a low socio-economic status*” (HS13). Infrastructure and resource limitations were perceived to be a significant influence in the adoption of Verily Connect, potentially amplifying other barriers for caregivers.

### 3.3. Inner Setting

#### 3.3.1. Culture

##### Alignment with Organisational Values and Goals

The purpose of Verily Connect aligned with the culture and values of the health services, which facilitated the implementation of the program. Health service staff spoke about its strong affiliation with organisational goals:

Our organisation’s culture is that knowledge is power, so we really try to empower carers to manage their lives by using all that’s available in community. So, I think in terms of Verily being a tool to do that, to be able to access information, that it fits quite well with our organisation’s beliefs and culture. (HS7)

Verily Connect was seen to augment health service resources: “*It’s just part of our business. It’s what we do every day. If people came and asked about services for carers or people with dementia, it would be one of the tools that we would give them*” (HS14). The close connection with health service organisational objectives was viewed as contributing to the sustainability of Verily Connect: “*I think it’s something we’ll continue to use; it matches in with our goals of engaging with community, connecting people together using that technology*” (HS11). In this respect, alignment of the program with organisational values and goals was perceived to not only facilitate the adoption of the program, but also indicate its potential for sustained use.

#### 3.3.2. Implementation Climate: Relative Priority

##### Staffing and Competing Priorities

A lack of dedicated staff to implement Verily Connect and the need to manage multiple competing priorities were perceived as barriers to implementation: “*[We needed] someone to continually manage it and drive it… it’s not our only project that we have to run, there are other things happening as well, so we don’t have that time to dedicate to just Verily*” (HS15). Competing organisational concerns were also identified a barrier to implementation: “*Partly it’s the capacity of the staff to take these onboard, and then partly it’s the challenge of meeting priorities in small health services where you’re trying to provide everything to everyone*”(HS1). In addition, changes to personnel created interruptions to the smooth implementation of Verily Connect: “*We had so many changes with the staff members… every time there was a change someone new had to hit the ground running*” (HS16). The lack of dedicated staff and competing organisational priorities were perceived by participants to result in the deprioritisation and disruption of the program, with limited capacity and resourcing issues as barriers to both the adoption and sustainability of the program.

#### 3.3.3. Readiness for Implementation: Leadership Engagement

##### Supportive Management

Support from management was considered an important factor in facilitating the implementation of Verily Connect: “*Our CEO is very focused on caring for older people and people with memory problems, so [manager] was a driver for that, for participation in the program*” (HS11). There was the view that, without management support, Verily Connect would not have been implemented: “*[Management] were very interested in it, or we wouldn’t have been told about it… they love their community, and they love their jobs, so if they weren’t interested it wouldn’t have happened*” (HS13). In this respect, leadership endorsement was viewed as a critical facilitator, with management buy-in acting as a powerful legitimiser of the program.

### 3.4. Individual Characteristics

#### 3.4.1. Knowledge and Beliefs About the Intervention

##### Preference for Face-to-Face Support

Negative or resistant views about online technology held by potential users were a barrier to implementation. In addition, older people had preferences for face-to-face support, which did not include support mediated by online technology: “*I think they just wanted to talk to someone about it, or meet with others who are going through it, or pick up the phone and talk to someone who is experiencing the same sort of difficulties they are*” (HS4). The staff attempted to accommodate caregivers’ preferences for existing methods of support. However, this approach worked against the uptake of Verily Connect: “*Going from feedback from clients who did want more of that face-to-face social connectedness, so [Verily Connect] did have, sort of like a bit of a backseat, because it’s all about what the community wants*” (HS8). However, trying to accommodate support provision through traditional methods was difficult to achieve: “*I think nothing will ever replace having that person one-to-one, speaking face-to-face… but it’s having those resources and the time and funding to be able to provide that support—that is very limiting*” (HS10). Participants recognised the tensions between caregivers’ preference for face-to-face interaction and community needs, and the ability of staff to meet that preference/those needs.

#### 3.4.2. Individual Stage of Change

##### Personal Readiness

Because a dementia diagnosis is life-altering for both the person living with dementia and caregiver, caregivers experienced a state of overwhelm which impacted their use of the application:

It was either too big a leap because it was not something that they were comfortable doing or using, or they didn’t have the time, brain space capacity at that point in time, because they were overwhelmed with looking after their own stuff at home, with the situation that they were in. (HS7)

The readiness of caregivers to adopt the program was influenced by the timing of the intervention in the dementia journey, and their emotional responses to a dementia diagnosis. A staff member reported that, “*It was difficult to recruit participants into the program, I think mainly because coming from the memory clinic side of things, so everything’s quite overwhelming for people when you’re having a diagnosis and that*” (HS11). Some staff relayed the preference of caregivers to have more traditional methods of communication: “*From our experience, that time after diagnosis can be a bit overwhelming and the journey’s a bit up and down, and often the face-to-face connection of being able to talk to somebody is what people look for*” (HS6). Verily Connect was designed to augment support for caregivers rather than replace existing services; however, some staff emphasised the preference of caregivers to speak with someone, particularly given their overwhelming situation:

For some people that’s [dementia diagnosis] very overwhelming and what they need is to engage in a two-way conversation with somebody around their concerns and what’s going to happen. Reading information sometimes doesn’t connect with the emotional things that people are going through. (HS6)

#### 3.4.3. Other Personal Attributes

##### Lack of Digital Literacy, Fear of and Resistance to Technology

A lack of older people’s experience using online technology was perceived to be a barrier to participating in Verily Connect: *“I think for those people who are not confident with technology, that will be the barrier”* (HS2). The staff considered that as a group, most older people *“were a little bit nervous about the technology*” (HS3), and lacked skills in using online technology: *“Unless they’re using it consistently or unless they’re tech savvy… I think they’re always worried that they’re going to break something if they hit the wrong button”* (HS4). Some staff perceived older people as having a generalised fear of, or resistance to, technology: *“We do have an ageing population, and they have this phobia of technology”* (HS8). Some staff thought that over time, fearful attitudes and reluctance to use new technology would abate:

It’s like any new technology; people are always a little bit suspicious… I think in a way it’s a bit like video consultations in general practice… we’re finding in our hospitals now that we’re doing a lot more remote consultations, so it is something that will happen. (HS3)

Some were hopeful that eventually there would be an uptake of Verily Connect: “*I think there is value in it. It just might take a few years for those people who are more able to engage with services through technology to take it up*” (HS6). In this respect, the program was viewed as being ‘ahead of its time’, suggesting that readiness was not only individual but also societal, with a mismatch between digital interventions and generational user attitudes or levels of digital literacy.

##### Privacy Concerns

Staff perceived that potential users could be put off from engaging with Verily Connect because they were worried about privacy issues: “*A lot of them are quite private and perhaps a little bit afraid to let other people know what they’re actually going through… I do find a lot of carers tend to don’t ask for help*” (HS5). Privacy is a significant concern in small rural communities, as a smaller population size can make individual activities more noticeable to others. The staff thought that potential users might be worried about being identified: “*[Name of rural town] isn’t that big a town, and they don’t want people to know their business*” (HS9). Similarly, another staff member stated that, “*They don’t want people to know that they’ve got someone that’s struggling*” (HS5). To some degree, privacy concerns in small rural communities acted as barriers to the uptake of the program due not only to the social dynamics of tightly knit communities, but also to the stigma around dementia and perceptions of caregiver stress.

### 3.5. Process

#### 3.5.1. Engaging

##### Challenges Recruiting Volunteers

Recruitment was considered a challenge due to the age of the caregiver and volunteer cohorts: “*Your target group were old people; I think all roads lead back to this*” (HS12). Specifically, technological aspects of Verily Connect were viewed as a barrier: “*Anything involving technology was unfortunately a bit of a negative to recruiting the volunteers*” (HS4). The stage of the dementia journey was also seen as contributing to a lack of participation, as was the busyness of prospective caregiver participants: “*Generally, people were interested, however, they just said that they were too busy and weren’t sure how they would find the time. That’s when it was really hard to sell the project*” (HS11). A lack of interest was also viewed as a challenge to recruitment: “*Talking to people, quite often they would commit to participating, or [to] talk to people about participating, but it didn’t seem to lead to anything. That was the challenge*” (HS1). Recruitment was constrained by perceived age-related barriers to technology, as well as the limited capacity among caregivers and volunteers to provide time to the program.

## 4. Discussion

The barriers to uptake identified in the current study are consistent with those reported in other studies of digital interventions, particularly those with rural and older adult populations. Digital literacy challenges are reported as enduring barriers among older caregivers, hindering effective engagement with digital or virtual solutions [39,40]. Similarly, other studies report that uptake of virtual support systems in dementia care is limited by low digital confidence and a preference for in-person interactions [41,42]. Compounding these challenges, rural telehealth implementation is frequently constrained by poor infrastructure, including limited internet access and the high cost of devices, which undermines equitable access to care [43]. Supportive management was an enabler of Verily Connect. Other studies have similarly demonstrated that committed leadership facilitates resource allocation and helps overcome systemic barriers to technology integration in care settings [41].

At the personal level, older caregivers faced challenges related to limited digital literacy and apprehension towards adopting unfamiliar technologies. Verily Connect aimed to address these issues by embedding volunteers to support the caregivers, and by using design features to ensure that it was easy to use for older people who were hesitant users of technology. Other authors have similarly suggested mitigating practices such as gradually introducing technology in parallel with existing, trusted forms of support to foster confidence and reduce resistance to change [39,40].

At the organisational level, the implementation of Verily Connect was supported by alignment with organisational goals and supportive management; other authors have similarly recommended that implementation efforts benefit from a clear internal structure and leadership [27,44]. Assigning dedicated roles, such as digital champions or implementation leads, can ensure consistent oversight, capacity building, and the embedding of new strategies into routine practice [45,46]. Strong leadership is also essential to ensure that local needs are met through a clearly defined digital strategy, which serves as a critical framework for coordinating activities and guiding capability-building initiatives [44]. Thus, consistent with our findings, having well-communicated strategies and strong alignment with broader organisational goals enhances overall readiness and fosters staff engagement across all levels of rural services.

Environmental or system-level barriers also play a significant role in rural and remote contexts, where limited internet connectivity and insufficient technological infrastructure hinder both the delivery and uptake of digital interventions. Addressing these challenges demands strategic investments in internet access, equipment, and technical support systems to create a digitally inclusive environment [43,47]. When the three domains of personal capacity, organisational readiness, and environmental infrastructure are addressed in tandem, the potential for virtual support tools to positively transform dementia care in rural settings is significantly enhanced.

The evaluation highlighted implementation challenges due to both individual and systemic factors, while enablers were primarily organisational. Our findings highlight the need for a dual approach, strengthening caregiver digital literacy and addressing infrastructure gaps, alongside fostering organisational readiness and leadership support. Co-design and integration into existing health service models appear critical for enhancing usability and sustainability of technology-based interventions in rural dementia care.

In terms of limitations, challenges in achieving data saturation may have arisen due to the small sample sizes of the key informant group. The small sample size itself may potentially reflect a selection bias, as participants who are more comfortable or experienced with digital solutions may have been more likely to volunteer to participate in the study. Furthermore, the subjective perspectives of health service professionals may not fully reflect the lived experiences and needs of caregivers. While the study involved sustained engagement with staff over an 18-month period, high staff turnover presented a limitation. This turnover meant that some staff had only brief involvement, and the perspectives of those who may have had deeper insights into implementation, but who left prior to the study, were not captured. Lastly, given the involvement of 12 communities from a specific region of Australia (south-eastern), the generalisability of findings to other rural or international settings may be limited.

Future research should build on the insights from this study to further optimise digital technology adoption in rural dementia care. As online communication becomes increasingly normalised, there is a need to develop more accessible solutions, such as platforms optimised for smartphone use. Targeted interventions aimed at improving digital literacy among older caregivers in rural settings are also essential. Additionally, longer-term studies are needed to track how attitudes toward digital support evolve over time. Notably, this study and intervention were conducted prior to the COVID-19 pandemic. Since then, older adults have demonstrated an increased comfort with and adoption of digital tools, with research showing a sustained use of online technologies and greater reliance on online platforms for health and social support [48,49,50]. This shift suggests that the appeal and relevance of digital solutions may be significantly greater in the post-pandemic context. In addition, evidence of the benefits of virtual memory cafés during the pandemic [51] further emphasises the potential value of technological interventions for caregivers of people living with dementia, warranting renewed investigation into their impact and uptake.

## Figures and Tables

**Table 1:** Overview of thematic findings.

CFIR Constructs	Themes	Barrier or Facilitator
**I. Innovation Characteristics**		
Evidence strength and quality	Grounded in research	Facilitators
Design quality and packaging	Supportive information and resources	
**II. Outer Setting**		
Needs and resources	Resource and infrastructure challenges	Barrier
**III. Inner Setting**		
Culture	Aligns with organisational values and goals	Facilitator
Implementation climate Relative priority	Staffing and competing priorities	Barrier
Readiness for implementation Leadership engagement	Supportive management	Facilitator
**IV. Individual Characteristics**		
Knowledge and beliefs about the intervention	Preference for face-to-face support	Barriers
Individual stage of change	Personal readiness	
Other personal attributes	Lack of digital literacy, fear of and resistance to technology Privacy concerns	
**V. Process**		
Engaging	Challenge recruiting volunteers	Barrier

## Data Availability

The original contributions presented in this study are included in the article. Further inquiries can be directed to the corresponding author.

## Abbreviations

The following abbreviations are used in this manuscript:

CFIR	Consolidated Framework for Implementation Research

## Appendix A. Interview Schedule

1. Please tell me about the activities you participated in as part of the VERILY project
2. How complicated was it to implement VERILY? Were changes or alterations made to the way that VERILY was implemented in your community?
3. What other carer support programs are available in your area? How does VERILY compare to other existing programs; does it replace or complement other programs? Is there another way of attaining support that carers would rather use? Do you think there was a strong need for VERILY?
4. How well do you think VERILY met the needs of your clients? Do you think VERILY was effective in your area?
5. What barriers did your clients experience with accessing VERILY?
6. What stories have you heard about the experiences of participants with VERILY?
7. How do you think your organisation’s culture (general beliefs, values, assumptions that people embrace) affected the implementation of VERILY? What was the general level of receptivity in your organisation to implementing VERILY?
8. How essential is VERILY to assisting you to meet the needs of your clients or your other organisational goals? Please describe the activities or initiatives that have the highest priority for your organisation? To what extent did VERILY take a backseat to other high-priority initiatives? How did you juggle completing priorities in your work?
9. To what extent does implementing VERILY provide an advantage to your town compared to other towns?
10. How well did VERILY fit with existing work processes and practices in your area? Can you describe how VERILY will be integrated into your ongoing work processes?
11. Did you have sufficient resources to implement VERILY? If yes, what was the quality of these resources?
12. What training did you receive in implementing VERILY? How prepared did you feel to use VERILY initiatives? Who did you ask if you had questions about VERILY or its implementation?
13. Were there incentives to help you ensure that the implementation of VERILY was successful?
14. What level of endorsement or support for VERILY did you see or hear from leaders? What level of involvement did leadership at your organisation have with VERILY? What kind of support or actions from leaders was needed to help VERILY be implemented?
15. Did you or others act as champions for VERILY? If yes, what behaviours or actions did you or others take to champion VERILY? What steps have been taken to encourage people to commit to using VERILY? What communication strategy was used to get the word out about VERILY?
16. Is there anything else that you would like to say about VERILY that we haven’t covered?

The questions were developed using the Consolidated Framework for Implementation Research Questions, which are available at: https://cfirguide.org/guide/app/#/guide_select (accessed on 20 July 2025).
